# Unexpected proximal ureteral stricture in a 5-year-old girl: case report

**DOI:** 10.1093/jscr/rjaf695

**Published:** 2025-08-29

**Authors:** Souha Qarouach, Jaouad Bouljrouf, Hadjar Nassiri, Ismail Benomar, Monim Ochan, Mounir Kisra

**Affiliations:** Faculté de Medecine et de Pharmacie de Rabat, Université Mohammed V de Rabat, Avenue Mohammed Belarbi El Alaoui, Souissi, Rabat 10170, Rabat, Morocco; Pediatric Surgery Rabat, Ibn Sina University Hospital Center, Rue Lamfadel Cherkaoui, BP 6527, Rabat 1000, Rabat-Sale-Zemmour-Zaer, Morocco; Faculté de Medecine et de Pharmacie de Rabat, Université Mohammed V de Rabat, Avenue Mohammed Belarbi El Alaoui, Souissi, Rabat 10170, Rabat, Morocco; Pediatric Surgery Rabat, Ibn Sina University Hospital Center, Rue Lamfadel Cherkaoui, BP 6527, Rabat 1000, Rabat-Sale-Zemmour-Zaer, Morocco; Faculté de Medecine et de Pharmacie de Rabat, Université Mohammed V de Rabat, Avenue Mohammed Belarbi El Alaoui, Souissi, Rabat 10170, Rabat, Morocco; Pediatric Surgery Rabat, Ibn Sina University Hospital Center, Rue Lamfadel Cherkaoui, BP 6527, Rabat 1000, Rabat-Sale-Zemmour-Zaer, Morocco; Laboratory of Life and Health Sciences, Faculty of Medicine and Pharmacy of Tangier Tetouan, Abdelmalek Essaadi University, Route Ziaten, BP 416, Tanger 90000, Tangier-Tetouan, Morocco; Faculté de Medecine et de Pharmacie de Rabat, Université Mohammed V de Rabat, Avenue Mohammed Belarbi El Alaoui, Souissi, Rabat 10170, Rabat, Morocco; Pediatric Surgery Rabat, Ibn Sina University Hospital Center, Rue Lamfadel Cherkaoui, BP 6527, Rabat 1000, Rabat-Sale-Zemmour-Zaer, Morocco; Faculté de Medecine et de Pharmacie de Rabat, Université Mohammed V de Rabat, Avenue Mohammed Belarbi El Alaoui, Souissi, Rabat 10170, Rabat, Morocco; Pediatric Surgery Rabat, Ibn Sina University Hospital Center, Rue Lamfadel Cherkaoui, BP 6527, Rabat 1000, Rabat-Sale-Zemmour-Zaer, Morocco; Faculté de Medecine et de Pharmacie de Rabat, Université Mohammed V de Rabat, Avenue Mohammed Belarbi El Alaoui, Souissi, Rabat 10170, Rabat, Morocco; Pediatric Surgery Rabat, Ibn Sina University Hospital Center, Rue Lamfadel Cherkaoui, BP 6527, Rabat 1000, Rabat-Sale-Zemmour-Zaer, Morocco

**Keywords:** stricture, ureteric, hydronephrosis, congenital

## Abstract

Congenital ureteral stricture is a rare but important differential diagnosis in antenatal hydronephrosis, often mistaken for ureteropelvic junction obstruction. We report the case of a 5-year-old girl who underwent surgery for suspected ureteropelvic junction obstruction based on imaging findings, including renal ultrasound and scintigraphy. Intraoperatively, the pyeloureteral junction appeared normal, but a proximal ureteral stricture was discovered 4 cm distal to the renal pelvis. Surgical management consisted of resecting the narrowed segment and performing a tension-free end-to-end ureteral anastomosis over a double-J stent. This case highlights the diagnostic limitations of standard imaging techniques in detecting ureteral anomalies and emphasizes the importance of accurate diagnosis and optimal management to preserve renal function.

## Introduction

Hydronephrosis is the most frequently detected fetal urinary tract anomaly on prenatal ultrasound, with an estimated incidence of 0.2%–2% of all pregnancies. Among the potential causes, ureteropelvic junction obstruction is the most common, accounting for 40%–60% of cases [1, 2].

In contrast, congenital ureteral stricture remains a rare cause of antenatal hydronephrosis and is often mistakenly diagnosed as ureteropelvic junction obstruction due to overlapping clinical features [3]. This anomaly can present as unilateral or bilateral atresia, of varying length, and can affect any segment of the ureter, although the distal portion is reported as the most commonly involved [4, 5].

We report the case of a young girl who underwent surgical management for a pelvi-ureteric junction obstruction, during which an unexpected proximal ureteral stricture was discovered intraoperatively.

## Case report

We report the case of a young girl who underwent surgical management for a pelvi-ureteric junction obstruction, during which an unexpected proximal ureteral stricture was undertaken to evaluate the underlying cause.

Renal ultrasound revealed a significant right-sided hydronephrosis with parenchymal thinning. The renal pelvis measured 56 mm in anteroposterior diameter, with a cortical thickness of 5 mm at the mid-renal level and 6 mm at the lower pole. The lumbar ureter appeared thin. These findings were suggestive of a ureteropelvic junction obstruction.

Dynamic renal scintigraphy (Tc-99m DTPA with furosemide challenge) showed a markedly prolonged and indeterminate renal transit time on the right side. There was significant pelvic dilatation and complete absence of drainage from the right kidney, even after furosemide administration.

**Figure 1:**
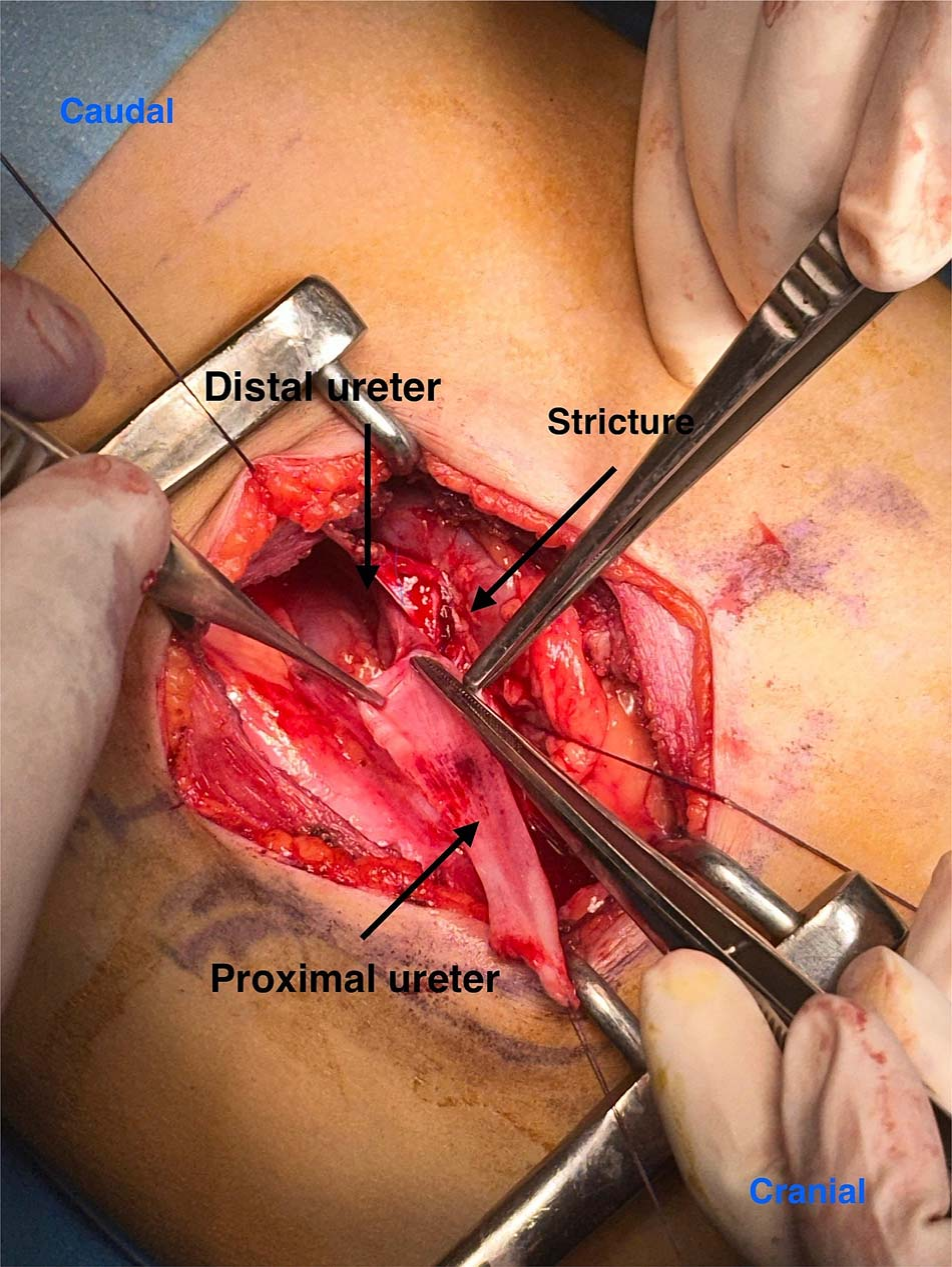
Intraoperative image showing a proximal ureteric stricture.

**Figure 2:**
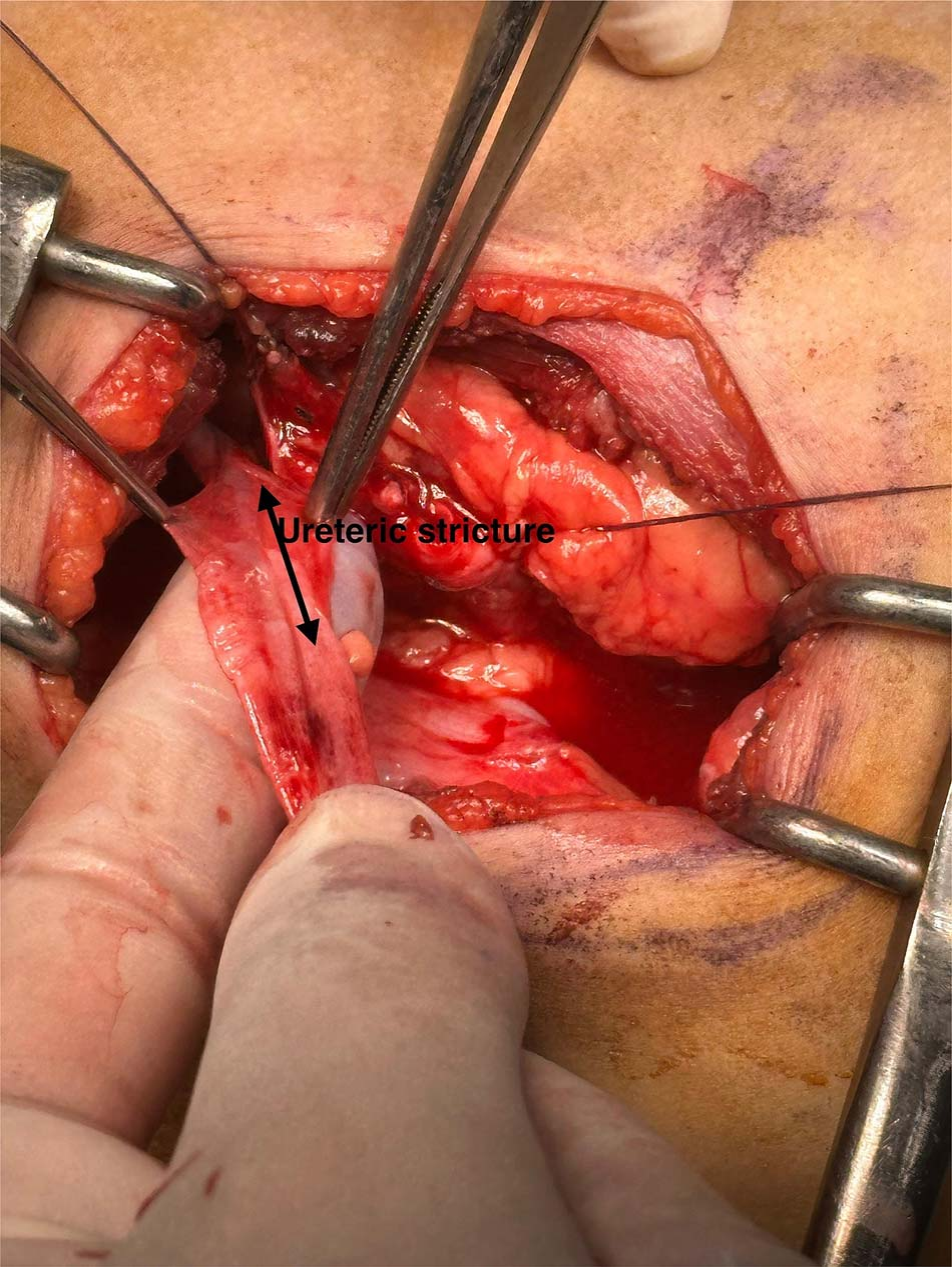
Intraoperative imaging shows a non-probe-patent mechanical obstruction of the proximal ureter, measuring 2 cm in length and located 4 cm below the ureteropelvic junction.

Given the suspicion of ureteropelvic junction obstruction and the impaired drainage, surgical intervention was planned. Intraoperatively, however, the ureteropelvic junction appeared normal. Further exploration revealed a proximal ureteral stricture located ~4 cm distal to the ureteropelvic junction (Fig. 1), extending over a 2 cm segment (Fig. 2).

The stenotic segment was resected, and a tension-free end-to-end (termino-terminal) ureteral anastomosis was performed. A double-J stent was placed to ensure proper drainage and healing.

## Discussion

Muscularization of the ureteral canal begins as early as the 12th week of gestation and continues throughout pregnancy. This developmental process may be disrupted by vascular compression, leading to incomplete formation of certain ureteral segments whether in the upper ureter, the mid-ureter at the pelvic brim, or the distal ureter near its insertion into the bladder. Such anomalies are often attributed to adjacent blood vessels, although these are not always visible on imaging. It is hypothesized that these vessels may regress during embryonic development, leaving behind a residual stricture [6].

Other mechanisms have also been proposed to explain the origin of congenital ureteral strictures. These include intrauterine ureteritis [7] and abnormal progression of the physiological process of ureteral occlusion followed by recanalization during fetal life [8]. Lastly, defective ureteral embryogenesis has been suggested, particularly due to the high incidence of associated genitourinary malformations [9].

Congenital ureteral strictures are rare and most commonly affect the distal portion of the ureter. In their discussions, Docimo *et al.* and Cauchi and Chandran highlighted this predominance by citing previously published series [3]. One such series reported that 74% of strictures involved the distal ureter, while only 6% were located in the proximal segment [10]. Isolated proximal ureteral strictures, such as the one observed in our case, remain exceptional and are often discovered incidentally during surgery.

Congenital ureteral stricture is a rare cause of antenatal hydronephrosis and is often misdiagnosed as ureteropelvic junction obstruction due to similar clinical presentations [3]. While most cases of antenatal hydronephrosis are managed conservatively with serial ultrasounds and tend to resolve spontaneously, strictures typically require surgical intervention to prevent progressive loss of renal function [11].

As highlighted by Hwang *et al.*, renal ultrasound and scintigraphy alone are often insufficient to pinpoint the exact location of the obstruction. Therefore, preoperative retrograde pyelography is recommended, especially when the distal ureter is not clearly visualized [12]. In uncertain cases, additional imaging such as magnetic resonance urography, computed tomography urography, or retrograde pyelography can help confirm the diagnosis. Our case illustrates the relevance of this approach, as retrograde pyelography might have enabled earlier identification of the stricture prior to surgical exploration.

The management of ureteral strictures typically involves surgical exploration, resection of the narrowed segment, and end-to-end anastomosis over a double-J stent, as was done in our case. However, in more complex or extensive strictures, this standard approach may not be sufficient. This is where the true surgical challenge lies: some situations require more advanced reconstructive techniques, such as interposition of an appendiceal or ileal segment, or grafting using bladder tissue or oral mucosa [13]. Alternatively, endourological procedures offer a less invasive option, often better tolerated and associated with quicker recovery, although they are not always suitable for severe cases [14].

This case underlines the importance of intraoperative vigilance. Even when the ureteropelvic junction appears normal, proximal ureteral segments should be examined, especially in the presence of unexplained hydronephrosis and thin ureter segments on imaging.

## Conclusion

Although rare, congenital ureteral strictures should always be considered in the differential diagnosis of antenatal hydronephrosis. A thorough understanding of this condition, combined with a careful and systematic radiologic evaluation, allows for accurate preoperative diagnosis and appropriate surgical planning.
